# Veterinary Legislation

**Published:** 1894-09

**Authors:** 


VETERINARY LEGISLATION.

Unusual activity is being manifested in the different States in securing legislation to *protect* the practitioner of veterinary medicine in the following of his vocation. Legislative interference in professional or business matters is a serious matter, and probably somewhat undemocratic. Any attempt to secure protection must be conducted in a liberal and diplomatic manner, or failure is sure to result, and justly so. There is an unwritten law in this country that every undertaking should stand or fall as its merits deserve, and protection should only be afforded when such protection is in the interests of the public at large. It is clearly evident, therefore, that any law intended to regulate the practice of medicine should be one in the nature of requiring information for the benefit of the public as to the qualifications possessed by the practitioners in a community, rather than one which compels the public to employ such persons as are considered by their colleagues as qualified to practice. Such a regulation of practice as would compel a man to state on his door-plate, and on his visiting cards, or whatever form of advertisement he might choose to use, whether he be qualified or unqualified, is, logically speaking, just what should be desired, and nothing more Every man should have a right to employ whomsoever he pleases to doctor himself or his animal, but the State should guarantee as qualified to perform such service only such persons as have shown a certain definite standard of proficiency. While this would be probably the fairest and best safeguard to the public, yet the members of our profession seem in most cases to look so unfavorably upon such a project, that legislation on this line has, so far as the writer knows, never been attempted. A sort of compromise measure is generally adopted, whereby all who possess any claim whatever to proficiency in the art of medicine, by reason of a certain number of years of practice, generally five, are allowed to register with those who are graduates, and are thereby guaranteed as *qualified* practitioners by the State. A provision is, however, always inserted, that on and after a certain date, only graduates may register. We do not, by any means, wish to make it appear that we are not favorable to veterinary legislation as at present practiced. Quite to the contrary, we wish to assist heartily in everything which will tend to make the profession better and of more value to the public. We simply wish to suggest that such legislation as would confine the word *qualified* to those practitioners who have graduated from a veterinary school, and who in addition have passed an examination before a State board, leaving the balance of the practitioners to follow their vocation as before, would place the profession on a better footing than will legislation which is essentially protection.

---

